# Optimizing Myocardial Protection in Minimally Invasive Cardiac Surgeries: A Network Comparison of Del Nido, Histidine-Tryptophan-Ketoglutarate, and Blood Cardioplegia

**DOI:** 10.3390/jcm13226977

**Published:** 2024-11-19

**Authors:** Sadeq Al-Hasan-Al-Saegh, Sho Takemoto, Stefano Benenati, Saeed Shafiei, Senol Yavuz, Mattia Galli, Florian Helms, Lukman Amanov, Nunzio Davide De Manna, Saeed Torabi, Jan Karsten, Jan Dieter Schmitto, Fabio Ius, Tim Kaufeld, Jawad Salman, Aron-Frederik Popov, Bastian Schmack, Arjang Ruhparwar, Alina Zubarevich, Alexander Weymann

**Affiliations:** 1 Department of Cardiothoracic, Transplantation and Vascular Surgery, Hannover Medical School, 30625 Hannover, Germanyweymann.alexander@gmail.com (A.W.); 2 Center for Transplantation Sciences, Department of Surgery, Massachusetts General Hospital and Harvard Medical School, Boston, MA 02114, USA; 3 Cardiovascular Disease Unit, IRCCS Ospedale Policlinico San Martino, IRCCS Italian Cardiology Network, 16132 Genova, Italy; 4 Department of Cardiac and Thoracic Vascular Surgery, Marburg University Hospital, 35043 Marburg, Germany; 5 Department of Cardiovascular Surgery, University of Health Sciences, Bursa Yuksek Ihtisas Training and Research Hospital, 16310 Bursa, Turkey; 6 Department of Cardiology, Maria Cecilia Hospital, GVM Care & Research, 48033 Cotignola, Italy; 7 Department of Anesthesiology, University Hospital Cologne, 50931 Cologne, Germany; 8 Department of Anaesthesiology and Intensive Care Medicine, Hannover Medical School, 30625 Hannover, Germany

**Keywords:** myocardial protection, cardioplegia, minimally invasive cardiac surgery, cardiac surgery, del Nido, histidine-tryptophan-ketoglutarate, blood cardioplegia, meta-analysis

## Abstract

**Background/Objectives**: The optimal choice of cardioplegia solution in minimally invasive cardiac surgeries (MICS) remains debated, as prolonged myocardial protection is essential to avoid interruptions to the surgical flow, which can prolong aortic cross-clamp time and cardiopulmonary bypass time, especially in the constrained surgical field. We conducted a network meta-analysis to evaluate the safety and efficacy of the del Nido (DN), histidine-tryptophan-ketoglutarate (HTK), blood cardioplegia (BC), and St. Thomas’ (STH) solutions in MICS. **Methods**: Medical electronic databases were thoroughly searched without time restrictions, including all types of studies except for study protocols and animal research. The final search was completed in June 2024. Subsequently, a network meta-regression was performed on both primary and secondary endpoints, utilizing R (The R Foundation for Statistical Computing, version 3.6.2) for the analysis. Meta-analyses were carried out using Review Manager software. **Results**: A total of 15 studies, enrolling 2282 patients, were included in the analysis. None of the comparisons showed statistically significant differences in in-hospital mortality between the four cardioplegia solutions (BC vs. HTK, OR: 3.21, 95% CI: 0.13–80.84; DN vs. HTK, OR: 1.42, 95% CI: 0.28–7.23; STH vs. HTK, OR: 1.25, 95% CI: 0.19–8.20). **Conclusions**: In this network meta-analysis of cardioplegia solutions in MICS, no significant differences were observed in major clinical outcomes across the solutions. Cardioplegia solutions that provide long-lasting myocardial protection with a single dose, such as DN and HTK, were found to be safely applied in MICS. DN was associated with shorter CPB times and HTK was associated with shorter hospital stays, though these differences may not have clinical implications.

## 1. Introduction

Cardiac arrest during cardiac surgery is typically achieved using cardioplegia under cardiopulmonary bypass (CPB), which provides a bloodless surgical field and myocardial protection [1]. Various cardioplegia formulations offer different durations of protection, myocardial preservation, and clinical outcomes [1]. Minimally invasive cardiac surgery (MICS) has gained increasing popularity due to its reduced invasiveness, blood loss, and need for transfusion, as well as shorter required hospital stays compared to conventional cardiac surgery [2,3]. However, the optimal choice of cardioplegia solution in MICS remains debated, as prolonged myocardial protection is essential to avoid interruptions to the surgical flow, which can prolong aortic cross-clamp time and CPB time, especially in the constrained surgical field.

Since the first clinical use of Bretschneider’s solution in the 1960s, various solutions—including crystalloid cardioplegia (CCP) solution, histidine-tryptophan-ketoglutarate (HTK) solution, St. Thomas’ (STH) solution, and blood cardioplegia (BC) solution, which combines Buckberg solution with oxygenated blood—have been widely used [1,4]. In MICS, where long-lasting myocardial protection with a single dose is preferred, the use of the del Nido (DN) cardioplegia solution, originally developed for pediatric cardiac surgery and containing lidocaine, has rapidly expanded alongside HTK [1,5].

While some studies have compared cardioplegia solutions head-to-head, results have often been inconsistent [1]. Moreover, there is limited data specifically focused on the MICS setting. To address these gaps in knowledge, we conducted a network meta-analysis to evaluate the safety and efficacy of DN, HTK, BC, and STH solutions in MICS. This analysis combined both direct and indirect evidence to provide clinical guidance on the optimal choice of cardioplegia solution in minimally invasive cardiac surgery.

## 2. Materials and Methods

### 2.1. Eligibility Criteria

This study was carried out following the Cochrane Collaboration published guidelines and the Preferred Reporting Items for Systematic Reviews and Meta-Analyses (PRISMA) guidelines (appendix) [6]. A network meta-analysis comparing the DN, HTK, BC, and STH solutions in MICS was conducted. The other part of this study was focused on providing reliable comparisons of outcomes and complications between the DN, HTK, BC, and STH solutions in two-group direct comparisons.

Therefore, the research question was structured using the Population, Intervention, Comparison, Outcome, and Study (PICOS) framework. Studies were included in the analysis based on the following criteria.

- Population: all patients underwent MICS
- Intervention: the DN, HTK, BC, and STH solutions as cardioplegia solutions
- Comparator: direct and indirect comparison with other solutions
- Outcome: postoperative clinical outcomes and complications
- Study design: Original articles were considered in the initial evaluation, while experimental studies, case reports, conference abstracts, letters, editorials, reviews, and general overviews were excluded.

### 2.2. Ethics

Since our study was based on existing literature and did not use information from human subjects, there were no ethical issues related to medical ethics that needed to be considered.

### 2.3. Literature Search

The Cochrane Library, Embase, PubMed, PubMed Central, OVID Medline, and Web of Science databases were systematically searched using a combination of the following search terms: “del Nido cardioplegia solution” OR “del Nido” OR “Bretschneider cardioplegic solution” OR “HTK solution” OR “Custodiol solution” OR “HTK solution of Bretschneider” OR “Bretschneider solution” OR “histidine-tryptophan-ketoglutarate solution” OR “St. Thomas’ Hospital cardioplegic solution” OR “STH solution” OR “cardioplegic solution STH” OR “Plegisol” OR “blood cardioplegia” OR “potassium cardioplegic solution” OR “crystalloid cardioplegic solution” OR “University of Wisconsin-lactobionate solution” OR “UW solution” OR “University of Wisconsin cardioplegic solution” OR “Cardiosol” OR “Celsior” AND “minimally invasive” OR “minimally invasive cardiac surgery” OR “cardiac surgery” OR “heart surgery”. There were no restrictions on the publication year during the literature search. The final search was conducted in June 2024. Furthermore, the reference lists of the retrieved articles were thoroughly examined to identify any additional relevant studies that might have been missed in the initial search.

### 2.4. Data Extraction

All titles and abstracts were screened independently by multiple authors (S.A.-H.-A.-S., S.S., S.Y., and S.T. (Saeed Torabi)). Eligible full-text articles were subsequently and independently reviewed by five authors (S.S., S.B., S.Y., S.T. (Sho Takemoto), and S.A.-H.-A.-S.) to determine inclusion and extract data. Any disagreements were resolved by the senior authors (A.Z. and A.W.). All data were compiled into an Excel table.

### 2.5. Outcome Measures

The following information was extracted from the included studies: first author’s name, year of publication, country of origin, sample size, study design, type of comparisons, propensity-matched design, demographic data and baseline characteristics of patients, procedural details, type of surgery, minimally invasive techniques, cardioplegia solution used, method of administering cardioplegia, dosage and volume of cardioplegia, duration of cardiopulmonary bypass (CPB), and aortic cross-clamping (ACC) time. Additionally, information on clinical outcomes and surgical complications following MICS was recorded. This included any instances of in-hospital mortality, durations of ACC and CPB, occurrences of new-onset atrial fibrillation (POAF), usage of intra-aortic balloon pumps (IABP), administration of inotropes, incidence of stroke, acute kidney injury (AKI) with need for dialysis, cases of low cardiac output syndrome, postoperative re-explorations, prolonged ventilation, use of defibrillators, and the length of stay in the intensive care unit (ICU) and hospital.

### 2.6. Statistical Analysis for Network Comparison

The impact of various cardioplegic solutions on primary and secondary endpoints was analyzed using a Bayesian meta-analysis with a random-effects model, which integrated both direct and indirect evidence while assuming consistency among the data. Odds ratios (ORs) and 95% credible intervals (CrIs) were calculated using a Markov chain Monte Carlo simulation. To evaluate publication bias, we utilized comparison-adjusted funnel plots of effect size versus standard error, along with Egger’s tests. Smaller studies often highlight treatment effectiveness, potentially skewing results in network meta-analyses due to their focus on limited cohorts. To explore this, treatments were ranked from most effective to least effective. The consistency of the evidence was assessed using the node-splitting technique. Finally, a network meta-regression was conducted on the primary endpoint, with the analysis performed using R (The R Foundation for Statistical Computing, version 3.6.2).

### 2.7. Statistical Analysis for Two-Group Comparison

Meta-analyses were conducted using Review Manager software (RevMan version 5.3.5; The Cochrane Collaboration, The Nordic Cochrane Center, Copenhagen, Denmark). Due to anticipated clinical heterogeneity among the included studies, the Mantel–Haenszel random-effects model was employed. Dichotomous data were reported as odds ratios (ORs), while continuous data were presented as weighted mean differences (MDs). Summary effect measures included the corresponding 95% confidence intervals (CIs). Statistical heterogeneity was assessed using the I^2^ statistic, where values between 0% and 25% indicated negligible heterogeneity, 26% and 50% indicated low heterogeneity, 51% and 75% indicated moderate heterogeneity, and 76% and 100% indicated high heterogeneity. A fixed-effects model was applied when the I^2^ was less than 50%, while a random-effects model was used for I^2^ values greater than 50%.

## 3. Results

**Figure 1:**
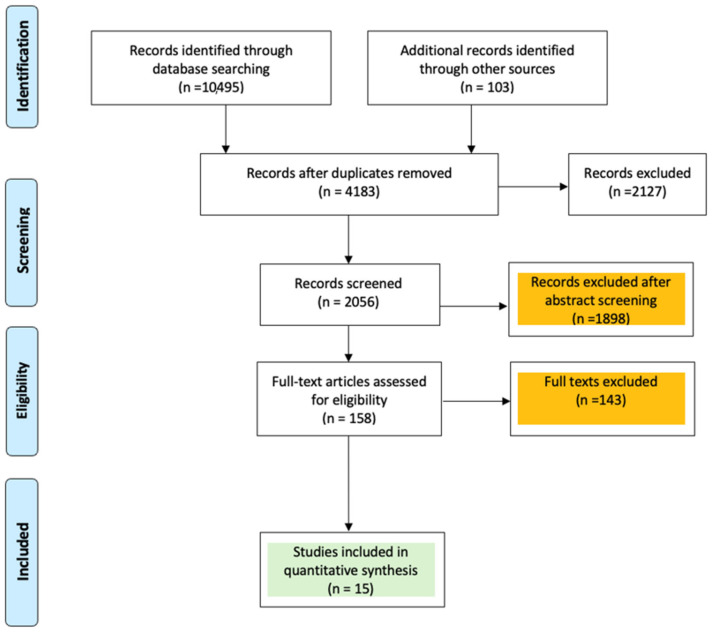
PRISMA flow diagram.

A total of 15 studies, enrolling 2282 patients, were included in the analysis [7,8,9,10,11,12,13,14,15,16,17,18,19,20,21] (Figure 1). Of these, two were prospective randomized studies, two were prospective nonrandomized studies, and eleven were retrospective observational studies, including ten propensity-matched studies (Table 1). The network meta-analysis involved comparisons between four cardioplegia solutions: DN vs. HTK (four studies), DN vs. BC (four studies), HTK vs. BC (four studies), HTK vs. STH (two studies), and DN vs. STH (one study). Detailed characteristics of the included studies, patients’ baseline data, and procedural details are presented in Supplemental Tables S1–S3.

### 3.1. Network Comparison

#### 3.1.1. Primary Endpoint (In-Hospital Mortality)

**Figure 2:**
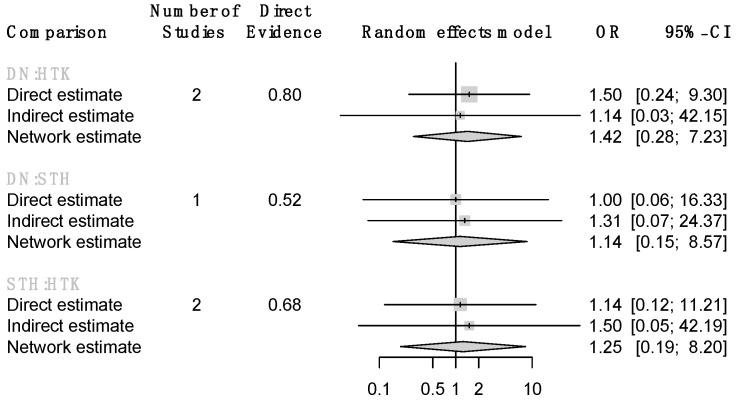
Forest plots for in-hospital mortality.

The results of the primary endpoint are displayed in Figure 2. None of the comparisons showed statistically significant differences in in-hospital mortality between the four cardioplegia solutions (BC vs. HTK—OR: 3.21, 95% CI: 0.13–80.84; DN vs. HTK—OR: 1.42, 95% CI: 0.28–7.23; STH vs. HTK—OR: 1.25, 95% CI: 0.19–8.20).

#### 3.1.2. Secondary Endpoints

**Figure 3:**
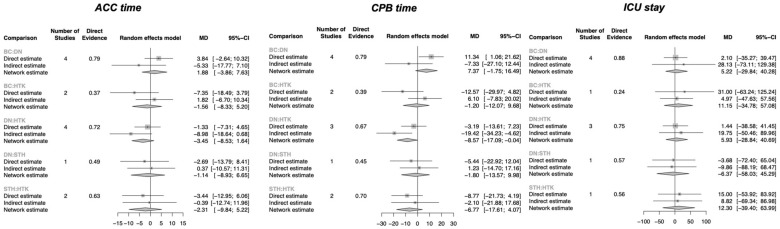
Forest plots for the secondary outcomes (ACC: aortic cross-clamping; CPB: cardiopulmonary bypass; ICU: intensive care unit).

There was no significant difference in ACC time across the cardioplegia solutions (Figure 3). However, DN was associated with a significantly shorter CPB time than HTK (MD: −8.57 min, 95% CI: −17.09–0.04) (Figure 3). No significant differences were observed in ICU stays between the groups (Figure 3).

**Figure 4:**
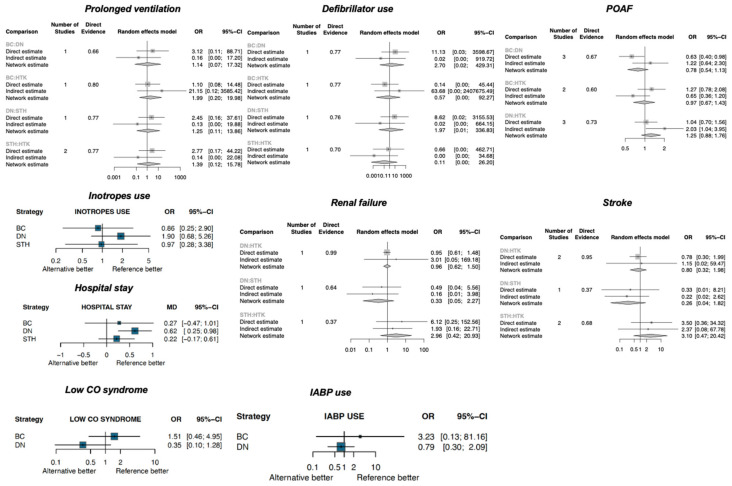
Forest plots for the secondary outcomes (continued) (POAF: postoperative atrial fibrillation; low CO syndrome: low cardiac output syndrome; IABP: intra-aortic balloon pump).

On the other hand, DN was associated with a longer hospital stay compared with HTK (MD: 0.62 days, 95% CI: 0.25–0.98) (Figure 4). There were no significant differences in inotrope use, IABP use, low CO syndrome, renal failure, stroke, prolonged ventilation, or defibrillator use (Figure 4).

**Figure 5:**
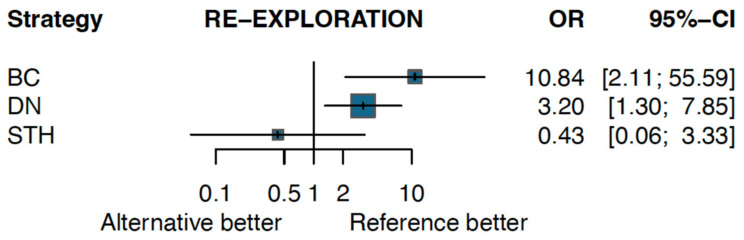
Forest plot for the incidence of re-exploration.

The odds of re-exploration were significantly higher for both the BC and DN solutions compared to HTK (BC vs. HTK—OR: 10.84, 95% CI: 2.11–55.59; DN vs. HTK—OR: 3.20, 95% CI: 1.30–7.85). In contrast, there was no significant difference in re-exploration between STH and HTK (OR: 0.43, 95% CI: 0.06–3.33) (Figure 5).

#### 3.1.3. Heterogeneity, Node-Split Analysis, and Publication Bias

**Figure 6:**
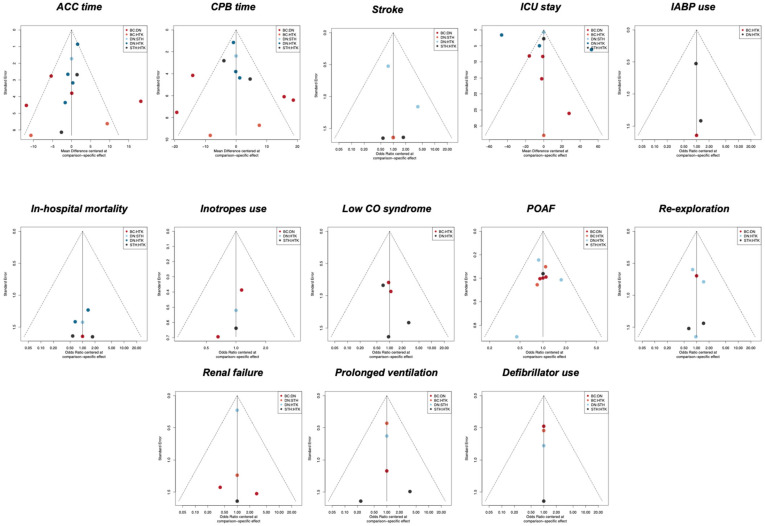
Comparison-adjusted funnel plots (ACC: aortic cross-clamping; CPB: cardiopulmonary bypass; ICU: intensive care unit; POAF: postoperative atrial fibrillation; low CO syndrome: low cardiac output syndrome; IABP: intra-aortic balloon pump).

Supplemental Table S4 displays the I^2^ statistics for individual comparisons for each endpoint, with heterogeneity ranging from low to high. Node-split did not show significant inconsistencies between direct and indirect evidence. Comparison-adjusted funnel plots were not suggestive of significant publication bias (Figure 6).

#### 3.1.4. Ranking the Strategies

**Figure 7:**
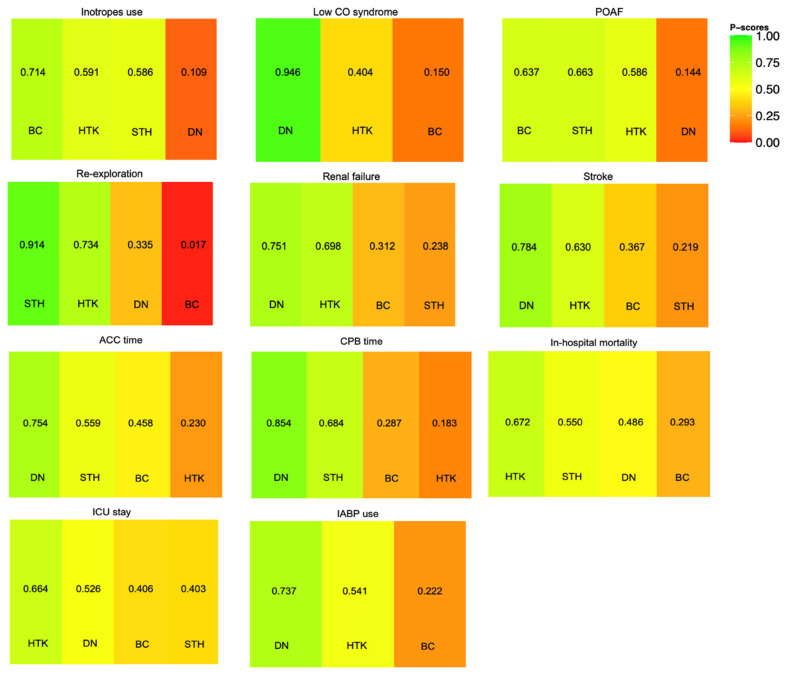
Rankogram of the four cardioplegia solutions for each outcome (ACC: aortic cross-clamping; CPB: cardiopulmonary bypass; ICU: intensive care unit; POAF: postoperative atrial fibrillation; low CO syndrome: low cardiac output syndrome; IABP: intra-aortic balloon pump).

Among the four solutions, the DN solution was ranked first for ACC time, CPB time, IABP use, low CO syndrome, renal failure, and stroke (Figure 7).

### 3.2. Two-Group Comparison

#### 3.2.1. Group 1: DN vs. HTK

**Figure 8:**
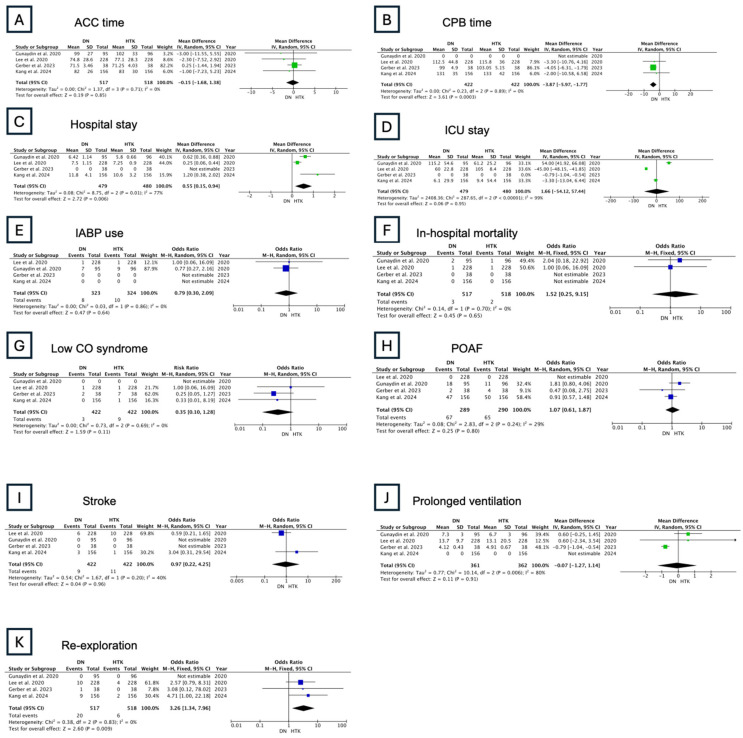
Results of the random-effects model: DN vs. HTK (ACC: aortic cross-clamping; CPB: cardiopulmonary bypass; ICU: intensive care unit; POAF: postoperative atrial fibrillation; low CO syndrome: low cardiac output syndrome; IABP: intra-aortic balloon pump) [7,8,9,10].

Early outcomes were reported in four studies, with a total of 517 patients. CPB time was significantly shorter in DN compared to HTK, while no significant difference was observed in ACC time (CPB time—MD: −3.87 min, 95% CI: −5.97–1.77; ACC time—MD: −0.15 min, 95% CI: −1.68–1.38; Figure 8). Significantly longer hospital stays and higher odds of re-exploration were observed in HTK (MD: 0.55 days, 95% CI: 0.15–0.94; OR: 3.26, 95% CI: 1.34–7.96, respectively; Figure 8). There were no significant differences in ICU stay, IABP use, in-hospital mortality, low CO syndrome, POAF, stroke, or prolonged ventilation (Figure 8).

#### 3.2.2. Group 2: DN vs. BC

**Figure 9:**
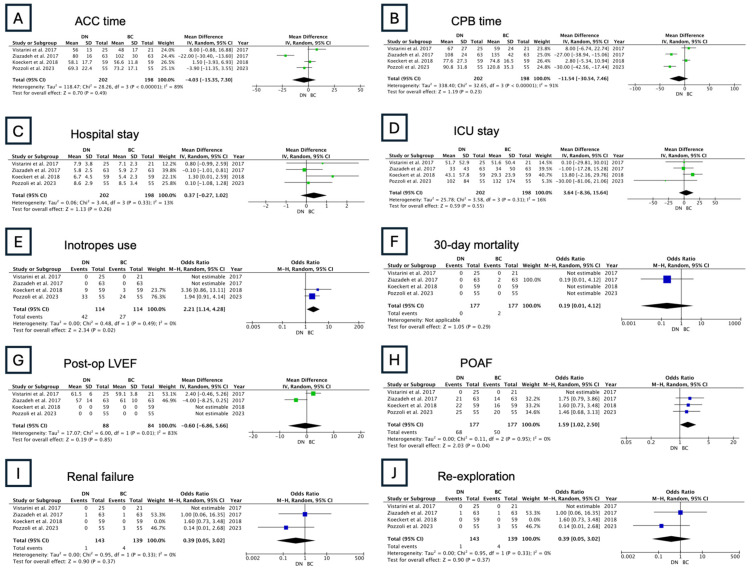
Results of the random-effects model: DN vs. BC (ACC: aortic cross-clamping; CPB: cardiopulmonary bypass; ICU: intensive care unit; POAF: postoperative atrial fibrillation; LVEF: left ventricle ejection fraction) [11,12,13,14].

Outcomes were reported in four studies, with a total of 202 patients. There were no significant differences in ACC time, CPB time, or 30-day mortality. The BC solution was associated with higher odds of inotrope use (OR: 2.21, 95% CI: 1.14–4.28; Figure 9), while there was no significant difference in postoperative LVEF. The BC solution was also associated with higher odds of POAF (OR: 1.59, 95% CI: 1.02–2.50; Figure 9).

#### 3.2.3. Group 3: HTK vs. BC

**Figure 10:**
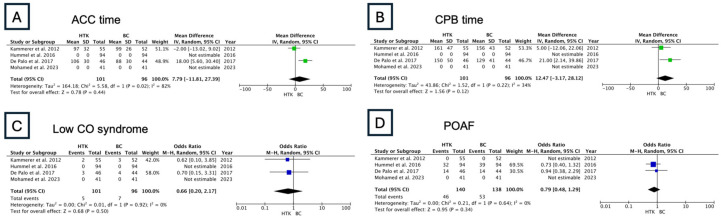
Results of the random-effects model: HTK vs. BC (ACC: aortic cross-clamping; CPB: cardiopulmonary bypass; POAF: postoperative atrial fibrillation; low CO syndrome: low cardiac output syndrome) [15,16,17,18].

Four studies and a total of 101 patients were included in the HTK vs. BC group. Both cardioplegia solutions were comparable with respect to ACC and CPB time, low CO syndrome, and POAF (Figure 10).

#### 3.2.4. Group 4: HTK vs. STH

**Figure 11:**
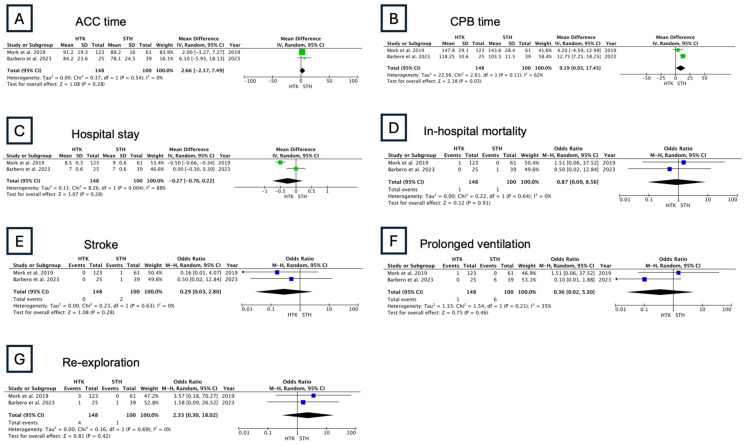
Results of the random-effects model: HTK vs. STH (ACC: aortic cross-clamping; CPB: cardiopulmonary bypass) [19,20].

CPB time was an only measured outcome that showed a significant difference, which was longer in HTK (MD: 9.19 min, 95% CI: 0.93–17.45; Figure 11). No significant differences were observed in ACC time, hospital stay, in-hospital mortality, stroke, prolonged ventilation, or re-exploration (Figure 11).

## 4. Discussion

In the present network meta-analysis, we evaluated the outcomes of different cardioplegia solutions, including DN, BC, HTK, and STH, across various clinical endpoints. The main findings of our analysis can be summarized as follows (1) DN was associated with a significantly shorter CPB time compared with HTK, although no significant differences were found in ACC time across the cardioplegia solutions; (2) DN was linked to a longer hospital stay compared with HTK, while no significant differences were observed in ICU stay between the groups; (3) re-exploration was significantly more frequent with both BC and DN compared to HTK, while STH showed no significant difference in this outcome; and (4) there were no significant differences among the cardioplegia solutions in terms of other key clinical outcomes, such as low cardiac output syndrome, renal failure, stroke, inotrope use, IABP use, prolonged ventilation, or defibrillator use.

HTK, known for its ability to provide myocardial protection for more than 120 min with a single dose, has been shown to offer comparable safety and myocardial protection to BC in randomized controlled trials (RCTs) [22,23]. The DN solution has gained rapid popularity in adult cardiac surgery following its initial use in pediatric cardiac surgery [5]. An RCT conducted by Talwar et al. reported that the DN solution demonstrated better preservation of the cardiac index, shorter ventilation times, shorter ICU and hospital stays, lower inotropic scores, reduced myocardial edema, and lower troponin-I release compared to HTK in pediatric cardiac surgery patients [24]. As of August 2024, only two RCTs compare the DN and HTK solutions in adult cardiac surgery, including one in MICS [8,25]. One of these RCTs, included in our analysis, reported comparable clinical outcomes with a single dose [8]. Still, the DN solution demonstrated a significantly higher incidence of atrioventricular block and longer ICU and hospital stays when comparing multiple doses of DN or HTK solutions [8]. Clinically small but statistically significantly shorter CPB time was consistent in our two-group comparison between the DN and HTK solutions.

In comparing the DN and BC solutions, no significant differences in perioperative outcomes were observed, except for a higher incidence of POAF in the DN solution among patients undergoing coronary artery bypass grafting with or without valve surgery [26], which was inversely observed in our two-group analysis of DN vs. BC (OR: 1.59, 95% CI: 1.02–2.50). However, there were no RCTs evaluating DN and BC specifically in MICS. Notably, an existing network meta-analysis that included all categories of patients and cardiac surgery types reported that the DN solution may be associated with lower perioperative mortality compared to the HTK or BC solutions, while the risk of POAF may be lower with HTK than with the BC or DN solutions in adults [1].

In contrast to the findings reported by Tan et al., both our network meta-analysis and two-group analysis did not demonstrate a mortality benefit with the DN solution compared to other cardioplegia solutions [1]. This discrepancy may be attributed to the fact that our analysis focused exclusively on MICS, which typically involves less complex surgical procedures.

In the context of MICS, minimizing interruptions during surgery is critical, particularly regarding cardioplegia administration. Repeated doses of cardioplegia can interrupt the flow of the surgical procedure and lead to prolonged ACC and CPB times. Therefore, a single dose of cardioplegia solution that provides prolonged myocardial protection is ideal. While Nagashima et al. reported the safety and efficacy of 60-min dosing intervals with a STH-based crystalloid cardioplegia solution, the DN solution can provide myocardial protection for up to 90 min and HTK for up to 120 min with a single dose [27]. In cases with no significant differences in major clinical outcomes, selecting a cardioplegia solution that offers longer myocardial protection is reasonable, although HTK showed a slightly longer CPB time than STH in the two-group comparison (MD: 9.19 min, 95% CI: 0.93–17.45), and DN vs. STH has not yet been well investigated. However, in cases where the ACC time exceeded 180 min, a comparison between nine patients who received DN and fifteen patients who received BC (with low-dose lidocaine) showed significantly higher postoperative CK-MB levels in the DN group (75.1 µg/L vs. 60.5 µg/L) [28]. These results suggest that the longer dosing interval associated with the DN solution may contribute to myocardial injury in cases with a prolonged ACC time. Therefore, in more complex and prolonged surgeries, it may be necessary to shorten the dosing interval of DN solutions to minimize the risk of myocardial damage.

In our results, although there was no significant difference in the ACC time between the cardioplegia solutions, CPB time was significantly shorter with the DN solution. However, many of the included studies involved relatively simple procedures that did not require prolonged ACC. Additionally, while statistically significant, the reported MD of −8.57 min in CPB time may not have a clinically meaningful impact. Similarly, while HTK was associated with a significantly shorter hospital stay compared to DN (MD: −0.62 days), the clinical relevance of this difference is limited.

One of the key features of the DN solution is the addition of lidocaine, a sodium channel blocker that suppresses membrane excitation. The membrane-stabilizing effect of lidocaine has been reported to result in a lower incidence of POAF and a reduced need for defibrillation after the release of the ACC [29,30,31,32]. However, in our analysis, no significant differences were observed in POAF or defibrillator use among the four groups. Regarding defibrillator use, each of the four comparison pairs was based on only one study. Therefore, further research is needed to investigate the restoration of spontaneous cardiac activity after ACC release and postoperative arrhythmia in MICS. In terms of re-exploration, the DN solution showed a statistically significant increase in re-exploration rates compared to HTK (OR: 3.2). Despite this finding, the absolute event counts were relatively low across studies (e.g., 10 re-explorations out of 228 in Lee et al. [9]), raising questions about the clinical relevance of this difference. Furthermore, MICS outcomes can be influenced by surgeon expertise and technique, which were not uniformly controlled across studies. Therefore, while the DN solution’s composition may provide some theoretical benefits in myocardial protection, its association with re-exploration requires validation through large, multi-center RCTs that account for surgical expertise, patient risk profiles, and procedural variables.

The significantly lower incidence of re-exploration observed with HTK in our analysis is unlikely to be attributed to the composition of the cardioplegia solutions, as there have been no reports suggesting that differences in cardioplegia formulations increase bleeding. Given that our study included multiple propensity-matched studies, differences in patient backgrounds and surgical procedures between groups were largely adjusted for. However, further investigation with larger sample sizes, standardized surgical techniques, and consistent surgical teams is required to validate this finding.

A systematic review and meta-analysis conducted by Chan et al. examined studies available in various databases up until April 2021. Given the increasing number of publications on minimally invasive cardiac surgery in recent years, an updated analysis was warranted, incorporating additional studies. Chan et al. found no significant differences between blood and crystalloid cardioplegia solutions in adult patients undergoing minimally invasive cardiac surgery. They concluded that the choice of cardioplegia solution in these procedures ultimately depends on the surgeon’s individual preferences and decisions [33]. Russell and colleagues highlighted that the selection of cardioplegia solution in MICS is primarily influenced by the surgeon’s individual preference. They found that solutions that enable longer cardiac arrest periods during infusion are often preferred, as they contribute to a more straightforward surgical process [34]. The study by Misra et al. did not identify any mortality benefits associated with the del Nido cardioplegia solution. However, they found that intraoperative glucose homeostasis was better maintained in the del Nido solution group. Additionally, this group experienced lower postoperative cardiac troponin T release and required fewer transfusions. The authors concluded that the del Nido cardioplegia solution is a safe and advantageous alternative to the blood cardioplegia solution in adult cardiac surgery [35].

There are several limitations to this study. First, the number of studies included in the network meta-analysis was relatively small, particularly for specific cardioplegia comparisons, which may have limited the statistical power of our findings. Second, most included studies were observational or propensity-matched rather than RCTs, which may introduce potential biases despite efforts to adjust for confounding factors. This lack of high-quality RCT data may impact the robustness and generalizability of our conclusions. Additionally, certain comparisons included small sample sizes, which further limited statistical power and our ability to detect clinically meaningful differences. Third, the heterogeneity in surgical techniques and endpoints across the included studies made it challenging to draw definitive conclusions. Finally, the lack of consistent reporting on specific outcomes, such as defibrillator use, further limited our ability to assess the full clinical impact of different cardioplegia solutions.

## 5. Conclusions

In this network meta-analysis of cardioplegia solutions in MICS, no significant differences were observed in major clinical outcomes across the solutions. Single-dose cardioplegia solutions such as the DN and HTK solutions were found to be safely applicable in MICS, with the DN solution associated with shorter CPB times and HTK with shorter hospital stays. Although these differences may not have substantial clinical implications, the findings suggest that both solutions provide effective myocardial protection in this context. Future research should focus on conducting larger, multi-center RCTs with standardized surgical protocols to validate these results. Additionally, exploring the use of single-dose cardioplegia solutions in more complex cardiac procedures may broaden the applicability of these findings. These directions could ultimately help to establish the optimal cardioplegia strategy for MICS and potentially extend its benefits to a wider range of cardiac surgeries.

## Figures and Tables

**Table 1:** List of included studies.

Author	Year	Study Period	Comparison	Country	Study Type	Propensity-Matched
Vistarini et al. [12]	2017	2012–2015	DN vs. BC	Canada	Single-center, retrospective, observational study	No
Lee et al. [9]	2020	2015–2019	DN vs. HTK	South Korea	Single-center, retrospective, comparative study	Yes
Ziazadeh et al. [14]	2017	2011–2016	DN vs. BC	USA	Single-center, retrospective, nonrandomized study	Yes
Luo et al. [21]	2019	2017	DN vs. STH	China	Single-center, retrospective, nonrandomized study	Yes
Mork et al. [19]	2019	2012–2019	HTK vs. STH	Switzerland	Single-center, retrospective, observational study	Yes
Barbero et al. [20]	2023	2014–2018	STH vs. HTK	Italy	Single-center, prospective, observational study	Yes
Mohamed et al. [15]	2023	2021–2022	HTK vs. BC	Egypt	Single-center, prospective, nonrandomized, observational study	No
De Palo et al. [17]	2017	2012–2015	HTK vs. BC	Italy	Single-center, retrospective study	No
Kang et al. [7]	2024	2018–2021	DN vs. HTK	Germany	Single-center, retrospective study	Yes
Gerber et al. [10]	2023	2016	DN vs. HTK	Poland	Single-center, retrospective, case-control study	Yes
Gunaydin et al. [8]	2020	2017–2019	DN vs. HTK	Turkey	Single-center, prospective, randomized study	No
Hummel et al. [16]	2016	2011–2015	BC vs. HTK	USA	Single-institution, retrospective, case-control study	Yes
Koeckert et al. [11]	2018	2013–2015	DN vs. BC	USA	Single-center, retrospective, nonrandomized study	Yes
Kammerer et al. [18]	2012	2008–2009	HTK vs. BC	Germany	Single-center, prospective, randomized study	No
Pozzoli et al. [13]	2023	2005–2015	DN vs. BC	Switzerland	Single-center, retrospective, observational study	Yes

Abbreviations: DN: del Nido solution; HTK: histidine-tryptophan-ketoglutarate solution; STH: St. Thomas’ solution; BC: blood cardioplegia solution.

## Data Availability

Data are available from the corresponding author upon reasonable request.

## Supplementary Materials

The following supporting information can be downloaded at: https://www.mdpi.com/article/10.3390/jcm13226977/s1, Supplemental Table S1: Baseline patients’ characteristics; Supplemental Table S2: Procedural characteristics; Supplemental Table S3: Procedural characteristics (continued); Supplemental Table S4: Heterogeneity across analyses.
