# Supplementary material for: Optimizing Myocardial Protection in Minimally Invasive Cardiac Surgeries: A Network Comparison of Del Nido, Histidine-Tryptophan-Ketoglutarate, and Blood Cardioplegia
Source: J Clin Med. 2024 Nov 19;13(22):6977. doi: 10.3390/jcm13226977 (PMC11594564; doi:10.3390/jcm13226977)
Supplement: Supplementary file 1 [file jcm-13-06977-s001.zip › jcm-3295414-supplementary.pdf]

Supplementary Appendix

Supplemental Table S1. Baseline patients' characteristics.

Author	Year	Solution	Sample Size (N)	Gender (N)	Male (%)	Mean Age (yrs)
**Vistarini et al.**	2017	DN	25	ND	ND	71±7
		BC	21	ND	ND	72±9
**Lee et al.**	2020	DN	228	113	49.6	59.8 ± 13.2
		HTK	228	123	53.9	60.3 ± 11.7
**Ziazadeh et al.**	2017	DN	63	41	65.1	66 ± 14
		BC	63	38	60.3	67 ± 3
**Luo et al.**	2019	DN	66	34	51.5	51.25 ± 5.05
		STH	66	35	53.0	51.68 ± 4.9
**Mork et al.**	2019	HTK	123	79	64.2	63.0 ± 11.5
		STH	61	39	63.9	64.2 ± 9.8
**Barbero et al.**	2023	STH	39	15	38.5	68.6 ± 13.4
		HTK	25	11	44.0	65.8 ± 15.3
**Mohamed et al.**	2023	HTK	41	28	68.3	52.05 ± 10.41
		BC	41	21	51.2	58.1 ± 5.15
**De Palo et al.**	2017	BC	44	21	47.7	54 ± 14
		HTK	46	24	52.2	59 ±14
**Kang et al.**	2024	DN	156	115	73.7	57±12
		HTK	156	110	70.5	57±12
**Gerber et al.**	2023	DN	38	31	81.6	50.5±5.4
		HTK	38	31	81.6	49.2±6.06
**Gunaydin et al.**	2020	DN	95	51	53.7	74±12
		HTK	96	58	60.4	71±14
**Hummel et al.**	2016	HTK	94	ND	ND	ND
		BC	94	ND	ND	ND
**Koeckert et al.**	2018	DN	59	46	78.0	69.1 ± 11.8
		BC	59	46	78.0	68.4 ± 10.8
**Kammerer et al.**	2012	HTK	55	30	54.5	65+14
		BC	52	35	67.3	66+9
**Pozzoli et al.**	2023	DN	55	39	70.9	68.8 ± 9.4
		BC	55	30	54.5	67.7 ± 11.7

Supplemental Table S2. Procedural characteristics.

Author	Year	Type of Surgery	Technique of Minimally invasive	Solution	Route	Cardioplegia dose
**Vistarini et al.**	2017	MI-AVR	J-Ministernotomy	DN	Anterograde	Single
				BC	Anterograde and/or Retrograde	Multiple
**Lee et al.**	2020	MICS (unspecified, including multiple procedures)	Right lateral minithoracotomy, right anterior minithoracotomy, or partial sternotomy (mainly upper)	DN	Anterograde	Single, an additional 500 mL was used when the cardiac ischemic time exceeded 100 minutes
				HTK	Anterograde	Single, an additional 1000 mL was used when the cardiac ischemic time exceeded 120 minutes
**Ziazadeh et al.**	2017	MI-AVR	Ministernotomy, right minithoracotomy	DN	Anterograde	Single
				BC	Anterograde and/or Retrograde	Multiple
**Luo et al.**	2019	MI-AVR, MVR, MVP, DVR	4th intercostal surgical incision with a thoracoscope (right anterolateral thoracotomy) or "L" suprasternal incision	DN	Anterograde	Single
				STH	Anterograde	Multiple
**Mork et al.**	2019	Full endoscopic MI-MVP	Right anterolateral minithoracotomy via the 4th intercostal space	HTK	Anterograde	Single
				STH	Anterograde	Single
**Barbero et al.**	2023	MI-MVP, TVP/TVR, ASD closure	Right mini-thoracotomy approach	STH	Retrograde	Single
				HTK	Retrograde	Single
**Mohamed et al.**	2023	MI-valve surgery (valve unspecified)	Right mini-thoracotomy approach	HTK	Anterograde	Single
				BC	Anterograde	Multiple
**De Palo et al.**	2017	MICS (MVR/MVP, TVP/TVR, myxoma, ASD closure, other combined surgery)	Right antero-lateral mini-thoracotomy, port-access video-assisted technique	BC	Antegrade (in case of Proplege use, 2/3 of the dose was administered antigradely and 1/3 retrogradely)	Multiple
				HTK	antegrade	Single
**Kang et al.**	2024	MI-MVP	Right minithoracotomy	DN	antegrade	Single
				HTK	antegrade	Single
**Gerber et al.**	2023	MI-MVP, plus ablation or LAA closure or PFO closure	Right mini-thoracotomy	DN	Antegrade	Single
				HTK	Antegrade	Single
**Gunaydin et al.**	2020	MI-AVR, plus aortic arch surgery or CABG or tricuspid valve surgery	Upper "J" ministernotomy, extention to the 3rd or 4th right intercostal space	DN	Antegrade	Single and/or Multiple
				HTK	Antegrade	Single and/or Multiple

**Hummel et al.**	2016	MI- and open valve surgery (AVR, MVR/MVP)	Right chest thoracotomy or partial sternotomy	HTK	Antegrade	Single and/or Multiple
				BC	Antegrade	Multiple
**Koeckert et al.**	2018	MI-AVR	Upper hemi-median sternotomy	DN	Antegrade 53, Retrograde 4, Both 2	Single
				BC	antegrade 20, retrograde 17, both 22	Multiple
**Kammerer et al.**	2012	MI-MVP	Right lateral minithoracotomy	HTK	Antegrade	Single
				BC	Antegrade	Multiple
**Pozzoli et al.**	2023	MI-MVR	Right minithoracotomy, Upper mini-sternotomy	DN	Antegrade	Single
				BC	Antegrade	Multiple

Supplemental Table S3. Procedural characteristics (continued).

Author	Year	Solution	Temprature	Volume of Cardioplegia	CPB Time (min)		ACC Time (min)		OP-Time (min)	
					Mean	SD	Mean	SD	Mean	SD
**Vistarini et al.**	2017	DN	ND	966±215 ml	67	27	56	13	ND	ND
		BC	ND	302±169 ml	59	24	48	17	ND	ND
**Lee et al.**	2020	DN	Systemic: 29ºC - 34ºC	1000 ml	112.5	44.8	74.8	28.6	ND	ND
		HTK	Systemic: 29ºC - 34ºC	2000 ml	115.8	36	77.1	28.3	ND	ND
**Ziazadeh et al.**	2017	DN	4ºC	1099 ± 342 ml	108	24	80	16	ND	ND
		BC	4ºC	4713 ± 1818 ml	135	42	102	30	ND	ND
**Luo et al.**	2019	DN	10ºC	1075 ± 86.6	100.81	15.08	58.81	10.9	ND	ND
		STH	10ºC	1568.75 ± 194.87	106.25	12.12	61.5	8.9	ND	ND
**Mork et al.**	2019	HTK	4ºC	20 mL/kg within 6 minutes	147.8	29.1	91.2	19.3	209.6	35.1
		STH	4ºC	30 mL/kg within 6 minutes	143.6	28.4	89.2	16	208	31.9
**Barbero et al.**	2023	STH	30ºC	1000 ml	105.5	11.55	78.1	24.5	ND	ND
		HTK	30ºC	25 ml/Kg	118.25	10.6	84.2	23.6	ND	ND
**Mohamed et al.**	2023	HTK	4ºC, Systemic: 28ºC	25 ml/kg is administered over 5-7 min	ND	ND	ND	ND	ND	ND
		BC	Systemic: 28-30ºC	1 L induction dose, 500 mL maintenance when repeated	ND	ND	ND	ND	ND	ND
**De Palo et al.**	2017	BC	34ºC	1 L induction dose, 500 mL maintenance when repeated	129	41	88	30	ND	ND
		HTK	32ºC	20 ml/kg in at least 6-8 minutes	150	50	106	30	ND	ND
**Kang et al.**	2024	DN	ND	1300	131	35	82	26	ND	ND
		HTK	ND	1850	133	42	83	30	ND	ND
**Gerber et al.**	2023	DN	4ºC	20 mL/kg maximal single dose: 1500 mL	99	4.9	71.5	3.46	ND	ND
		HTK	4ºC	1500 mL for patients <70 kg, 2000 mL for patients ≥70 kg	103.05	5.15	71.25	4.03	ND	ND
**Gunaydin et al.**	2020	DN	4ºC - 8ºC	2250±70	ND	ND	99	27	ND	ND
		HTK	4ºC - 8ºC	2400±80	ND	ND	102	33	ND	ND
**Hummel et al.**	2016	HTK	ND	2 L followed by redosing of 300 to 500 mL as needed after 2 hours	ND	ND	ND	ND	ND	ND
		BC	ND	Initial 1 L antegrade bolus, then redosed retrograde every 20 to 30 minutes	ND	ND	ND	ND	ND	ND
**Koeckert et al.**	2018	DN	ND	1290 ± 347	77.6	27.3	58.1	17.7	ND	ND
		BC	ND	2284 ± 828	74.8	16.5	56.6	11.8	ND	ND
**Kammerer et al.**	2012	HTK	4ºC	One bolus of 2000 mL	161	47	97	32	ND	ND

		BC	33ºC	2000 mL initial, readministration every 20 minutes	156	43	99	26	ND	ND
**Pozzoli et al.**	2023	DN	8ºC – 12ºC	1028±271	90.8	31.8	69.3	22.4	195	41.6
		BC	4ºC	476±123	120.8	35.3	73.2	17.1	225.1	45.1

Supplemental Table S4. Heterogeneity across analyses.

Endpoint	I^2 DN vs.HTK	I^2 DN vs. BC	I^2 HTK vs. BC	I^2 HTK vs. STH
**ACC time**	0	89	82	0
**CPB time**	0	91	34	62
**Hospital stay**	77	13	-	88
**ICU stay**	99	16	-	-
**IABP use**	0	-	-	-
**In-hospital mortality**	0	-	-	0
**Inotropes use**	-	0	-	-
**Low CO syndrome**	0	-	0	-
**POAF**	29	0	0	-
**Re-exploration**	0	-	-	0
**Renal failure**	-	0	-	0
**Stroke**	40	-	-	0
**Prolonged ventilation**	80	-	-	35
**Defibrillator use**	-	-	-	-

References

1. Kang, J.; Hoyer, A.; Dieterlen, M.T.; Oetzel, H.; Otto, W.; Ginther, A.; Pfannmüller, B.; Misfeld, M.; Noack, T.; Kiefer, P.; et al. Comparison of modified Del Nido and Custodiol® cardioplegia in minimally invasive mitral valve surgery. *Eur. J. Cardiothorac. Surg.* **2024**, *65*, ezae161. https://doi.org/10.1093/ejcts/ezae161.
2. Gunaydin, S.; Akbay, E.; Gunertem, O.E.; McCusker, K.; Kunt, A.T.; Onur, M.A.; Ozisik, K. Comparative Effects of Single-Dose Cardioplegic Solutions Especially in Repeated Doses During Minimally Invasive Aortic Valve Surgery. *Innov. Technol. Tech. Cardiothorac. Vasc. Surg.* **2021**, *16*, 80–89. https://doi.org/10.1177/1556984520967119.
3. Lee C hoon Kwon, Y.; Park, S.J.; Lee, J.W.; Kim, J.B. Comparison of del Nido and histidine-tryptophan-ketoglutarate cardioplegic solutions in minimally invasive cardiac surgery. *J. Thorac. Cardiovasc. Surg.* **2022**, *164*, e161–e171. https://doi.org/10.1016/j.jtcvs.2020.11.163.
4. Gerber, W.; Sanetra, K.; Gerber, A.D.; Jankowska-Sanetra, J.; Kuczera, M.; Białek, K.; Buszman, P.P.; Bochenek, A. One-shot cardioplegia for minimally invasive mitral valve repair-a comparison of del Nido and Bretschneider Histidine-Tryptophan-Ketoglutarate solutions. *Perfusion.* **2023**, *38*, 763–770. https://doi.org/10.1177/02676591221080653.
5. Koeckert, M.S.; Smith, D.E.; Vining, P.F.; Ranganath, N.K.; Beaulieu, T.; Loulmet, D.F.; Zias, E.; Galloway, A.C.; Grossi, E.A. Del Nido cardioplegia for minimally invasive aortic valve replacement. *J. Card. Surg.* **2018**, *33*, 64–68. https://doi.org/10.1111/jocs.13536.
6. Vistarini, N.; Laliberté, E.; Beauchamp, P.; Bouhout, I.; Lamarche, Y.; Cartier, R.; Carrier, M.; Perrault, L.; Bouchard, D.; El-Hamamsy, I.; et al. Del Nido cardioplegia in the setting of minimally invasive aortic valve surgery. *Perfusion.* **2017**, *32*, 112–117. https://doi.org/10.1177/0267659116662701.
7. Pozzoli, A.; Torre, T.; Bagnato, P.; Gallo, M.; Toto, F.; Ferrari, E.; Demertzis, S. del Nido versus hematic cardioplegia in minimally invasive aortic valve surgery. *Asian Cardiovasc. Thorac. Ann.* **2023**, *31*, 795–801. https://doi.org/10.1177/02184923231209858.
8. Ziazadeh, D.; Mater, R.; Himelhoch, B.; Borgman, A.; Parker, J.L.; Willekes, C.L.; Timek, T.A. Single-dose del Nido Cardioplegia in Minimally Invasive Aortic Valve Surgery. *Semin. Thorac. Cardiovasc. Surg.* **2017**, *29*, 471–476. https://doi.org/10.1053/j.semtcvs.2017.10.001.
9. Mohamed, M.N.; Shady, M.A.M.I.; Fadala, M.A.E.; Moustafa, M.G.E.D. Custodiol over cold blood cardioplegia in minimal invasive cardiac valve surgery. *Egypt. J. Surg.* **2023**, *42*, 554–558. https://doi.org/10.4103/ejs.ejs_110_23.
10. Hummel, B.W.; Buss, R.W.; DiGiorgi, P.L.; Laviano, B.N.; Yaeger, N.A.; Lucas, M.L.; Comas, G.M. Myocardial Protection and Financial Considerations of Custodiol Cardioplegia in Minimally Invasive and Open Valve Surgery. *Innovations* **2016**, *11*, 420–424.
11. De Palo, M.; Guida, P.; Mastro, F.; Nanna, D.; Quagliara, T.A.; Rociola, R.; Lionetti, G.; Paparella, D. Myocardial protection during minimally invasive cardiac surgery through right mini-thoracotomy. *Perfusion.* **2017**, *32*, 245–252. https://doi.org/10.1177/0267659116679249.

12. Kammerer, I.; Nagib, R.; Hipp, G.; Premar, M.; Hansen, M.; Franke, U. Myocardial Protection in Minimally Invasive Mitral Valve Surgery: Comparison of the Cold-Blood Cardioplegia of the Bretschneider Solution and the Warm-Blood Cardioplegia of the Calafiore Protocol. *Arch. Clin. Exp. Surg. ACES.* **2012**, *1*, 14. https://doi.org/10.5455/aces.20120220022958.
13. Mork, C.; Koechlin, L.; Schaeffer, T.; Schoemig, L.; Zenklusen, U.; Gahl, B.; Reuthebuch, O.; Eckstein, F.S.; Grapow, M.T.R. Bretschneider (Custodiol®) and St. Thomas 2 Cardioplegia Solution in Mitral Valve Repair via Anterolateral Right Thoracotomy: A Propensity-Modelled Comparison. *Mediat. Inflamm.* **2019**, *2019*, 5648051. https://doi.org/10.1155/2019/5648051.
14. Barbero, C.; Pocar, M.; Marchetto, G.; Cura Stura, E.; Calia, C.; Dalbesio, B.; Filippini, C.; Salizzoni, S.; Boffini, M.; Rinaldi , M.; et al. Single-Dose St. Thomas Versus Custodiol® Cardioplegia for Right Mini-thoracotomy Mitral Valve Surgery. *J. Cardiovasc. Transl. Res.* **2023**, *16*, 192–198. https://doi.org/10.1007/s12265-022-10296-z.
15. Luo, H.; Qi, X.; Shi, H.; Zhao, H.; Liu, C.; Chen, H.; Peng, R.; Yu, Z.; Hu, K.; Wang, C.; et al. Single-dose del Nido cardioplegia used in adult minimally invasive valve surgery. *J. Thorac. Dis.* **2019**, *11*, 2373–2382. https://doi.org/10.21037/jtd.2019.05.78.
